# New Insights into the Mammalian Egg Zona Pellucida

**DOI:** 10.3390/ijms22063276

**Published:** 2021-03-23

**Authors:** Carla Moros-Nicolás, Pascale Chevret, María Jiménez-Movilla, Blanca Algarra, Paula Cots-Rodríguez, Leopoldo González-Brusi, Manuel Avilés, Mª José Izquierdo-Rico

**Affiliations:** 1 Department of Cell Biology and Histology, Faculty of Medicine and Nursing, University of Murcia, Campus Mare Nostrum and IMIB-Arrixaca, 30100 Murcia, Spain; carla.moros@um.es (C.M.-N.); mariajm@um.es (M.J.-M.); b.algarraonate@um.es (B.A.); paula.cotsr@um.es (P.C.-R.); leopoldo.gonzalez@um.es (L.G.-B.); maviles@um.es (M.A.); 2 Laboratoire de Biométrie et Biologie Evolutive, UMR5558, CNRS, Université Claude Bernard Lyon 1, Université de Lyon, 69100 Villeurbanne, France; pascale.chevret@univ-lyon1.fr

**Keywords:** zona pellucida, ZP, pseudogenization, composition, molecular evolution, mammals

## Abstract

Mammalian oocytes are surrounded by an extracellular coat called the zona pellucida (ZP), which, from an evolutionary point of view, is the most ancient of the coats that envelope vertebrate oocytes and conceptuses. This matrix separates the oocyte from cumulus cells and is responsible for species-specific recognition between gametes, preventing polyspermy and protecting the preimplantation embryo. The ZP is a dynamic structure that shows different properties before and after fertilization. Until very recently, mammalian ZP was believed to be composed of only three glycoproteins, ZP1, ZP2 and ZP3, as first described in mouse. However, studies have revealed that this composition is not necessarily applicable to other mammals. Such differences can be explained by an analysis of the molecular evolution of the ZP gene family, during which ZP genes have suffered pseudogenization and duplication events that have resulted in differing models of ZP protein composition. The many discoveries made in recent years related to ZP composition and evolution suggest that a compilation would be useful. Moreover, this review analyses ZP biosynthesis, the role of each ZP protein in different mammalian species and how these proteins may interact among themselves and with other proteins present in the oviductal lumen.

## 1. Introduction

The zona pellucida (ZP) is an extracellular matrix that is synthesized during follicular development and finally surrounds the plasma membrane of the oocyte and preimplantation embryo of mammals. ZP functions are related to important events that take place during oocyte formation, fertilization, and early embryo development. The ZP is involved in folliculogenesis, granulosa cells organization and differentiation, recognition of and binding to the spermatozoa, induction of the acrosome reaction, blocks to polyspermy, and the protection of the preimplantated embryo [1,2,3,4,5,6,7,8,9,10,11].

The ZP forms a spherical shell that exhibits such properties as elasticity and porosity. The thickness varies greatly between different species, from being very thin in marsupials (ranging from 2 to 6 μm, depending on the species) to the ~1–25 μm of eutherian mammals [12]. In monotremes, the ZP is even thinner (0.5 μm around the time of ovulation), although it appears thicker during oogenesis [13].

Apparently uniform when visualized with an optic microscope, the ZP can be seen to be divided into different regions if analyzed by other methods. These regions could be related with differences in sperm binding capacity, and previous studies have pointed to a substantial difference in the ability to bind sperm in the outer region compared to the inner region of the ZP [7,14,15].

**Figure 1:**
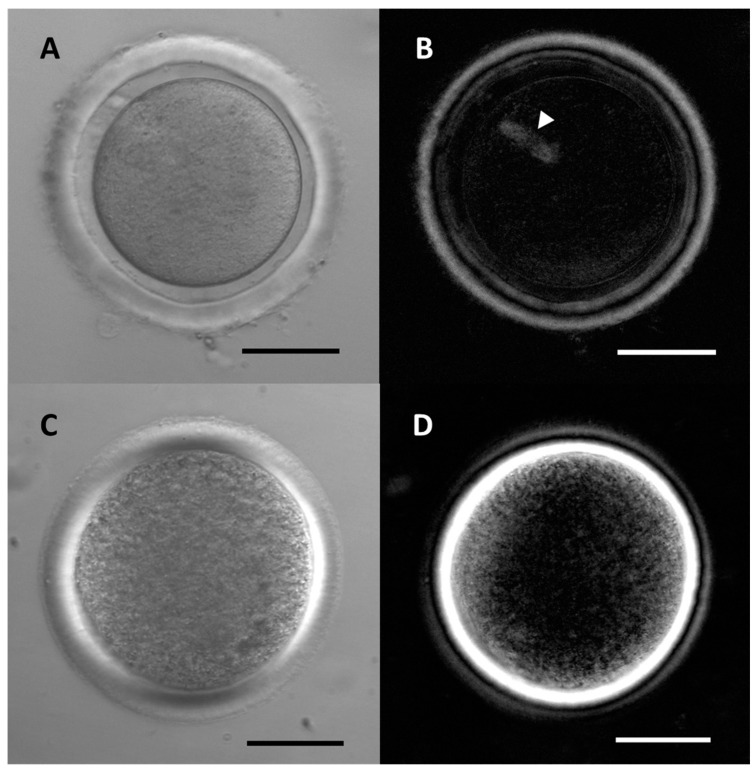
The zona pellucida (ZP) of rabbit (**A**) and bovine (**C**) oocytes captured by conventional Hoffmann inverted microscopy appears very similar. The same zona pellucida captured by polarized light microscopy (**B**,**D**) reveals a different multilaminar structure and distinctly birefringence, and the meiotic spindle is easily appreciated (arrowhead). Scale bars 50 µm.

By using a polarization microscope (PolScope) it was possible to observe that the structure of the ZP is more complex than was first thought, and is multilaminar [16]. Thus, several layers can be differentiated on the basis of their birefringence properties. Studies made by our group showed that rabbit and cattle ZP presented three layers. In rabbit, the inner and outer layers presented a higher degree of birefringence than the intermediate region, which showed no birefringence. In cattle, the inner region presented the highest degree of birefringence followed by the outer region, whereas the intermediate region showed little or no birefringence (Figure 1).

The molecular structure responsible for this multilayered structure and an explanation of how these differences may be involved in the differing ability to bind to sperm are still unknown. The use of more complex microscopy techniques such as the scanning electron microscope showed that the most compact ZP region was closer to the oocyte. In contrast, the most porous ZP region was in contact with the cells of the cumulus oophorus, and was the region involved in the sperm binding process [17].

The ZP matrix is formed by several glycoproteins, which interact to form cross-linked fibrils. The tridimensional architecture is known only in mouse, where the fibrils are polymers constructed of ZP2-ZP3 dimers and the long fibrils are interconnected by ZP1, which incorporates into ZP filaments using its ZP domain [18,19]. Fibrils in the inner and outer layers of the ZP are oriented perpendicular and parallel, respectively, to the oolemma, whereas fibrils in the intermediate layer are oriented randomly [16,20,21,22].

The proteins that form the ZP matrix are glycosylated. These specific oligosaccharide chain residues have been related to sperm receptor activity [6,23,24,25,26]. Lectin histochemistry was used in the identification and characterization of the carbohydrates present in ovarian follicles, as well as in pre- and post-fertilized oocytes in a wide range of species. Using this technique combined with enzymatic digestion and different chemical treatments, many authors suggested a heterogeneous glycoconjugate composition for the ZP [27,28]. This heterogeneity was described as being characteristic of several species and to change throughout follicular development. The results of an exhaustive revision of these differences can be consulted in the supplementary files (see Tables S1 and S2).

The house mouse (*Mus musculus*) was used as a model to study the structure and function of ZP for more than 35 years. Early studies in mouse demonstrated that the ZP in this species is formed of only three glycoproteins: Zp1, Zp2, and Zp3 [29], while *Zp4* is a pseudogene [30,31,32]. However, over the years, this composition was shown to only apply to mouse. Different events during ZP evolution explain the different models of ZP. This topic is explained in the following section.

## 2. ZP Composition Is Explained by Molecular Evolution

### 2.1. Origin of Vertebrate ZP Genes

Studies on the evolution of the ZP genes in vertebrates greatly benefitted from the increase in molecular data and complete genomes available. Using a phylogenetic approach and analyzing the cDNA sequences available in Genbank, Spargo and Hope divided the different ZP genes of vertebrates into four subfamilies (named *ZPA*, *ZPB*, *ZPC*, and *ZPX*) [33]. They proposed that at least one ZP gene evolved during the earliest stages of vertebrate evolution, probably before the divergence of fish and amphibians. This ancestral ZP gene gave rise to four different subfamilies as a result of at least three duplication events [33]. Some of these genes were later lost in some lineages and remain present only in fish or amphibians. Goudet et al. (2008) established a more complete list of vertebrate ZP genes, and classified them into at least six subfamilies: *ZP1*, *ZP2*, *ZP3*, *ZP4*, *ZPAX*, and *ZPD*. Phylogenetic analysis recovered two separate trees, the first one with the genes of the *ZP1*, *ZP2*, *ZP4*, *ZPD*, and *ZPAX* subfamilies and the second one with only genes from the *ZP3* subfamily. These two groups of genes might have had different ancestral genes. In the first tree, the *ZPD* subfamily is at the base of the phylogeny before *ZPAX* diverge, followed by *ZP2* and finally *ZP4* and *ZP1*. In most mammals, the authors identified a smaller number of genes due to several losses. Most placental mammals included in their analysis have four ZP proteins (ZP1, ZP2, ZP3, ZP4) and some of them only three, in the absence of ZP1 (dog or cattle) or ZP4 (*Mus musculus*) [32]. ZPD was found only in amphibians and ZPAX in amphibians and chicken, indicating that these two subfamilies were probably lost before the origin of mammals [32]. In a study made in our lab, we identified three ZP3-related genes in marsupials, which were named *ZP3-a*, *ZP3-b*, and *ZP3-c* [34]. The presence of this duplication in *ZP3* gene further complicates the models of ZP composition. In a recent study, Feng et al. (2018) identified eight ZP gene subfamilies (*ZP1/4*, *ZP2*, *ZPAX*, *ZPY*, *ZPD*, *ZP3-1*, *ZP3-2*, and *ZP3-3*), which were grouped into three clusters. The first cluster grouped *ZP1/4*, *ZP2*, *ZPAX*, and *ZPY*, the second one contained only ZPD and the third *ZP3-1*, *ZP3-2*, and *ZP3-3* [35]. A phylogenetic analysis of the two ZP3 datasets [26,27] indicated that our ZP3-a, b, and c sequences belong to the ZP3-1 subfamily of Feng et al. (2018) and should be named ZP3-1a, 1b, and 1c (Figure S1). Based on their phylogenetic analysis, Feng et al. also suggested that their three clusters have separate origins, the three ancestral genes expanding into eight subfamilies during the evolution of vertebrates [35]. The number of ZP genes in present-day vertebrates is the results of this complex evolution characterized by lineage-specific duplication and/or gene loss.

### 2.2. Mammalian ZP Genes

**Figure 2:**
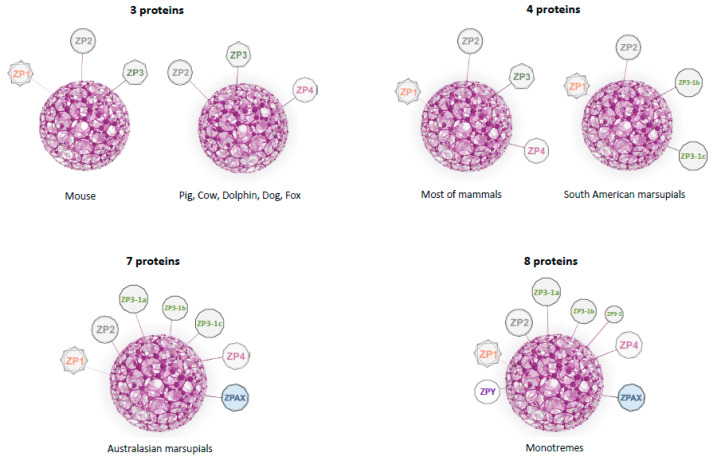
Zona pellucida composition in mammals. The ZP is composed of three, four, seven, or eight proteins. In house mouse (*Mus musculus*), the ZP is composed by ZP1, ZP2, and ZP3. In pig, cow, dolphin, dog, and fox, the ZP is formed by ZP2, ZP3, and ZP4. In most of mammals, the ZP is formed by four proteins: ZP1, ZP2, ZP3, and ZP4. In marsupials, two scenarios are found: four proteins in South American marsupials (ZP1, ZP2, ZP3-1b, and ZP3-1c) or seven proteins in Australasian marsupials (ZP1, ZP2, ZP3-1a, ZP3-1b, ZP3-1c, ZP4, ZPAX). In monotremes, the ZP is composed of eight ZP proteins: ZPY, ZP1 ZP2, ZP3-1a, ZP3-1b, ZP3-2, ZP4, ZPAX. In eutherian mammals ZP3-1c is written as ZP3 in order not to complicate the current nomenclature.

**Figure 3:**
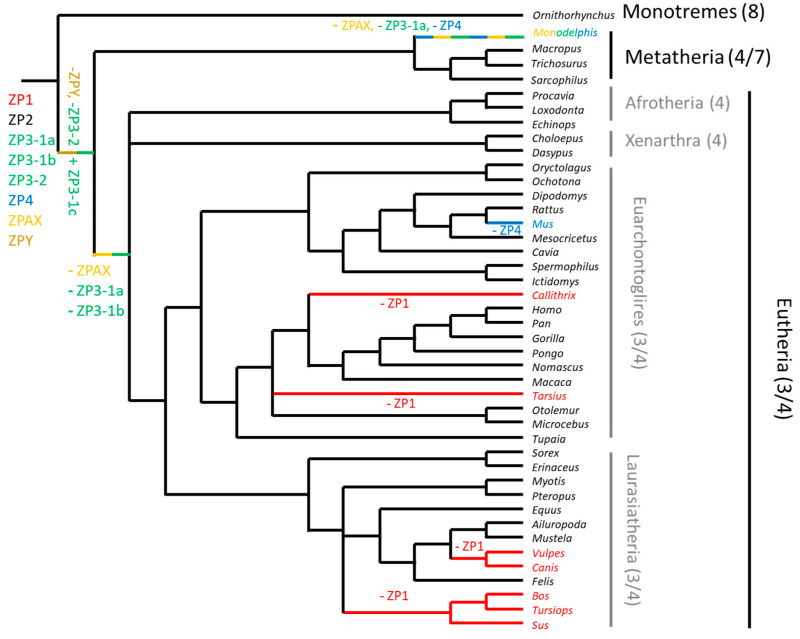
Evolution of ZP genes in mammals. Six ZP genes were probably present in the common ancestor. The gain and the loss of a gene in the phylogeny are indicated respectively by a plus or minus sign with the name of the gene concerned.

As well as by bioinformatic analysis, the presence of functional ZP genes in mammals was confirmed by mRNA sequencing and, in several species by proteomic analysis (e.g., human [30], hamster [36], rabbit [37], and cat [38]). The number of ZP genes in mammals varies from three to eight [29,32,34,37,38,39,40] (Figure 2). Recently, Feng et al. (2018) identified 13 ZP genes in the genome of *Ornithorhynchus* belonging to seven subfamilies (*ZP1*, *ZP2, ZP3-1*, *ZP3-2*, *ZP4*, *ZPAX*, and *ZPY)* [35]. Hence the ZP composition of *Ornithorhynchus* needs further studies to confirm this very high number of genes, especially the presence of the newly described *ZPY* and *ZP3-2* subfamilies [35]. Different phylogenetic studies indicated the presence of eight ZP subfamilies in the last common ancestor of present-day mammals: *ZP1*, *ZP2*, two ZP3-related subfamilies, named here *ZP3-1a* and *1b*, *ZP3-2*, *ZP4*, *ZPAX*, and *ZPY*. These eight subfamilies are present in the *Ornithorhynchus* [27] and *Tachyglossus* genomes (unpublished data). Duplication of one of the *ZP3* genes took place in the last common ancestor of placentals and marsupials (Figure 3) [34,39]. The genome of Australian marsupials contained up to seven ZP genes (*ZP1*, *ZP2*, three ZP3-related subfamilies, named here *ZP3-1a*, *1b* and *1c*, *ZP4*, and *ZPAX*), but several genes were lost in South American marsupials, e.g., the *Monodelphis* lineage, where *ZPAX*, *ZP3-1a*, and *ZP4* are pseudogenes [34]. The presence of a functional *ZPAX* in Australian marsupials seems to have only been confirmed in the case of *Macropus eugenii* [39] and *Trichosurus vulpecula* (XM_036748819), although the genomic data available for two other marsupials (*Vombatus ursinus* and *Phascolarctos cinereus*) suggests the presence of stop codons (unpublished data). Our molecular analysis of *ZPAX* gene expression in Bennett’s wallaby (*Macropus rufogriseus*) ovaries using reverse transcription–polymerase chain reaction (RT-PCR) showed no amplification of this gene (Figure S2 and Table S3). Whatever the case, additional studies are needed to confirm the presence of a functional *ZPAX* in one or several lineages of marsupials. Two of the three subfamilies of *ZP3* genes and *ZPAX* were lost in placental mammals. Evidence of *ZPAX* pseudogenes were found in human, chimpanzee, rhesus macaque, and cow [32]. Most placental mammals have retained four functional ZP genes, but some lineages have lost one ZP gene during their evolution. In some species, the *ZP1* gene has been lost (Figure 3), as first evidenced in *Canis* and *Bos* [32], in addition, *ZP1* pseudogenes were also found in *Tursiops* and *Sus* [37], indicating that *ZP1* pseudogenization took place early in the evolution of Cetartiodactyls. *ZP1* pseudogenes were also found in two primates (*Tarsius* and *Callithrix*, in two separate events) [37] and several carnivores (*Canis*, *Vulpes*, *Arctocephalus*, *Leptonychotes*, in three separate events) [40]. Thus, *ZP1* pseudogenization took place several times independently during the evolution of placentals (Figure 3). By contrast, *ZP4* was lost only once, in rodents belonging to subgenus *Mus* [41]. This subgenus was particularly well studied as it includes *Mus musculus*, the house mouse, whose ZP composition has been known for many years [29]. This genus comprises four subgenera (*Mus*, *Coelomys*, *Nannomys*, and *Pyromys*) and *Zp4* remains functional in three of them. The pseudogenization took place recently, less than 6 million years ago (Mya) when the *Mus* subgenus diverged from the three other subgenera [41]. In summary, although the ZP composition in present day mammals is derived from eight ancestral ZP genes, most mammals have four proteins (Figure 2). During the evolution of mammals one new gene arose through duplication of one of the two *ZP3* genes and several genes were independently lost (*ZPAX*, *ZPY*, *ZP3-1a*, *ZP3-1b*, *ZP1*, and *ZP4*) (Figure 3).

## 3. In Most Mammals There Are Four Proteins, but for What?

Functional data on the mammalian ZP proteins are derived from knock out (KO) studies in animals, especially the house mouse, which lacks Zp4 [29,30,31,32]. Targeted mutagenesis of endogenous mouse genes has provided substantial information on ZP functions. In this species, Zp1 is thought to offer stability and structural integrity to the matrix, since a KO mouse lacking this protein has an atypical ZP, which is more porous than normal [42]. Nevertheless, these mice are able to form a ZP that allows fertilization and the development of a normal preimplantation embryo, although litters are smaller than usual [42]. On the other hand, Zp2 and Zp3 proteins are essential for fertilization and embryo development [43,44,45]. *Zp2* null mice have a thinner ZP, which is lost at the end of the folliculogenesis [45], whereas in *Zp3* null mice, the ZP is not formed [43,44]. However, mouse ZP development is possible with only two glycoproteins (Zp1-Zp3 or Zp2-Zp3), the presence of Zp3 always being necessary [46]. Because *Zp4* is a pseudogene in this species [29,30,31,32], the role of this protein remained unsolved until very recently. However, rabbit ZP is composed of four glycoproteins [37], and our group has created the first female rabbit lacking *ZP4*, demonstrating that this protein is crucial for embryo development but not for fertilization [47]. The ZP of these females was more permeable, thinner, and exhibited a more disorganized and fenestrated structure, which suggests a structural role [47]. Furthermore, a recent study, also made in our lab, of heterologous in vitro fertilization between mouse species with three ZP proteins and species with four ZP proteins demonstrated that a ZP formed of four glycoproteins is not a barrier for the spermatozoa of species with a ZP formed of three glycoproteins [41].

Based on the results obtained from experiments with mice, Zp3 was long considered the primary sperm receptor (binding to the capacitated non-reacted sperm) [2,48], with Zp2 acting as secondary sperm receptor (binding to the capacitated and acrosome-reacted sperm) [49]. However, some years ago, it was demonstrated that the non-reacted sperm bind to the ZP2 N-terminal domain. This protein is modified after fertilization, losing its ability to bind to the sperm and inducing the blockage to polyspermy [50]. Other studies, using transgenic mice with hZP4, showed that human sperm do not bind to this humanized ZP, and that other ZP proteins are needed for human sperm recognition and binding [51]. One year later, Baibakov et al. created a transgenic mouse with hZP2 and demonstrated that human sperm only binds to the ZP, when it expresses ZP2, alone or is co-expressed with other ZP glycoproteins [52]. Other studies performed in human species with native and recombinant ZP proteins expressed in baculovirus and animal cells showed that ZP1, ZP3, and ZP4 bind to capacitated sperm inducing the acrosome reaction. However, when these glycoproteins are expressed in *E. coli*, there is no evidence of an acrosome reaction, perhaps due to the lower glycosylation pattern expressed in this system [9,53,54,55,56,57,58,59,60,61,62,63]. It was also reported that the ZP domain of *ZP1* is capable of inducing the acrosome reaction on its own [54].

Nevertheless, the actual functions of ZP proteins in a ZP composed of four glycoproteins are not well documented, except as regards the data available for rabbit lacking *ZP4* [47], although, data about ZP gene sequence variations in women provide some information. A compilation of ZP1, ZP2, and ZP3 mutations in women can be found in [22]. Recent studies indicated that *ZP1* mutations are related with infertility, which may be associated with empty follicle syndrome (EFS) [64,65,66,67,68,69] or with ZP-free oocytes [70,71,72,73]. It was suggested that *ZP1* mutations may affect the shuttling of glycoproteins to the secretory pathway, which would prevent the formation of the ZP around the oocyte, but also the formation and development of eggs [70]. However, it was recently suggested that this lack of ZP is due to an impairment of ZP1 secretion that leads to the absence of filament crosslinking [73,74]. On the other hand, *ZP2* mutations produce ZP-free oocytes [72] or oocytes surrounded by a thin ZP [68,72,75], as observed in KO mice for *Zp2* [45]. The mutated ZP2 proteins might not be secreted to the surface of the oocytes, leading to the formation of a thin and defective ZP that only consists of the other three ZP proteins [72]. These oocytes could not be fertilized by in vitro fertilization (IVF) techniques, but they were fertilized by intracytoplasmic sperm injection (ICSI) [75], which suggests that ZP2 is involved in sperm interaction and is essential for fertilization. In other studies, *ZP3* mutations, which induce zona free-oocytes [72,73] or EFS were described [73,76]. These mutations could impede ZP assembly, inducing oocyte degeneration and empty COCs [73,76]. In relation to *ZP4*, Wei et al., described some infertile patients with a thin and irregular ZP and identified some *ZP4* mutations; the authors proposing ICSI as a good strategy for fertilizing the oocytes [77].

Human and mouse studies indicated that the results obtained in one species cannot be extrapolated to others, as seen in the case of *Zp1* rescue mice and human *ZP1* mutations. Mice lacking *Zp1* form a ZP and are fertile [42], whereas human *ZP1* is an essential protein of the zona matrix [64,65,66,67,68,69,70,71,72,73]. This indicates that in species with four proteins the generation of KO animals is necessary in order to understand the functionality of the different ZPs, as already demonstrated in rabbits [47].

## 4. Biosynthesis of Zona Pellucida: Cellular Trafficking and Processing of ZP Proteins

The extracellular matrix ZP is assembled during oogenesis as the oocyte grows embedded in follicular structures guests in the ovary. There are many controversial questions about the synthesis and formation of the ZP, beginning with the cellular origin of the ZP proteins. Among mammals, the proteins are synthesized solely by the ovary, whereas among non-mammals they are synthesized either by the liver or the ovary, or by both organs [78]. The only species in which ZP glycoproteins were seen to be synthesized exclusively in the oocyte are mouse [21,79,80,81,82], rat [83], and hamster [84]. In most species, the granulosa cells and the oocyte participate in the ZP synthesis. Several studies performed in different animal species supported the hypothesis that the expression of the ZP during oogenesis is species-specific [85,86]. For example, rabbit ZP1 is expressed by oocyte and granulosa cells [87] and the same occurs for ZP3 in bovine and ZP4 in pig [86,88]. In the case of primates, both the oocyte and follicular cells express ZP2, ZP3, and ZP4 [85]. In human, the cell type responsible for synthesis of the ZP glycoproteins is controversial. According to some authors the ZP proteins are synthesized exclusively by the oocyte [89] or both oocyte and granulosa cells surround it [90,91,92]. A recent study, based on an analysis of folliculogenesis transcriptomics data [93] revealed expression of the four ZP genes in oocytes and follicular cells in all the stages (primordial, primary, secondary, antral, and pre-ovulatory). The only exception is ZP1, whose levels are not above 1 FPKM (fragments per kilobase million) (taken as baseline expression threshold) in 8 out of 18 primordial oocytes. ZP3 is the most expressed gene of all, while levels of ZP2 and ZP4 are lower and ZP1 is the least expressed gene. For the granulosa cells, levels of ZP1 mRNAs above 1 FPKM are only observed in secondary follicles. ZP2 and ZP4 are detected in low levels above 1 FPKM in primordial, primary, and secondary oocytes, but their median expression is null in larger follicles. ZP3 mRNA levels are considerably higher than those of the other ZP genes, with moderate levels from primordial to antral follicles, and low but above 1 FPKM in pre-ovulatory follicles. According to differential expression analysis, no statistically significant differences were observed for the ZP genes both for oocytes and granulosa cells of the 5 analyzed stages of follicular development.

At cellular level, the expression of ZP proteins and formation of the mammalian extracellular ZP began after follicular recruitment, when the oocyte enters a growth phase whereby it increases in volume, and undergoes replication and redistribution of the cytoplasmic organelles. Thus, ZP synthesis is a biochemical marker of follicular growth. To be active, ZP-domain proteins require a complex series of post-translational modifications, starting with the formation of intramolecular disulfides and the covalent addition of various sugar molecules, so that proteins must cross and circulate through the endomembrane system toward the cell surface. Consequently, these processes have to be highly regulated and coordinated in all components of the matrix to ensure proper protein folding and avoid premature polymerization. Interestingly, the observation that several ZP-domain proteins are targeted to distinct subapical regions of the plasma membrane provides evidence that the routing of the ZP-domain proteins to specific membrane subdomains probably involves highly regulated trafficking [94]. In addition, the extracellular protein forming the matrix has to be processed in at both extremities; a cleavage site for a signal peptide has been confirmed at the N-terminal by MS [95], and C-terminal with transmembrane domain and cytoplasmic tail must be released at this endoproteinase cleavage site to allow N-terminal ectodomain to be incorporated in the zona matrix [95,96,97]. Here, we summarize the cellular aspects of the growing oocytes coordinated by molecular trafficking and processing of the ZP proteins to form the extracellular matrix.

**Figure 4:**
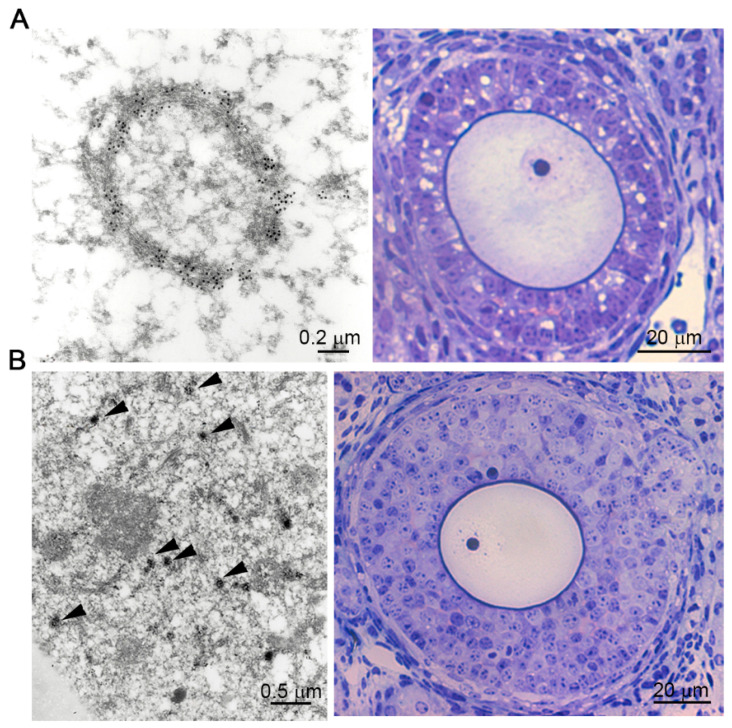
Ultrastructural analysis of the endoplasmic reticulum in the mice oocytes during the folliculogenesis. (**A**) Bilaminar primary ovarian follicle, semithin sections (right panel) and transmission electron microscope (panel left) immunostaining with anti-PDI antibody. The circular structure was specifically labelled with the anti-PDI antibody. (**B**) Multilaminar primary ovarian follicle, semithin sections (right panel), and transmission electron microscope (left panel) immunostaining with anti-PDI antibody. Note the presence of small dark vesicles (arrows) distributed throughout ooplasm specifically labelled with ER marker (Jiménez-Movilla M, Avilés M, Castells MT, Ballesta. Ultrastructural analysis of the endoplasmic reticulum in the mice oocytes during the folliculogenesis. First International Congress of Histology and Tissue Engineering, Spain, 2005).

The immunodetection of endogenous ZP proteins showed that ZP2 and ZP3 colocalize in unusual spherical structures (~2 µm), which can be immunostained by an antibody to the resident ER-chaperonin protein, protein disulfide isomerase (PDI), only in growing oocytes encased within bilaminar ovarian follicles in mice [98]. The endoplasmic reticulum (ER) undergoes a high degree of remodeling according to the requirements of the oocyte. In immature hamster oocytes, several large (8.4 ± 1.4 µm) surface masses were detected, which, subsequently, in mature MII-arrested oocytes, disaggregate and form many smaller clusters throughout much of the ooplasm, thereby generating a finely organized network of accumulated peripheral ER [99]. Using ultrastructural immunogold cytochemistry analysis of mouse ovaries, elongated cisternae studded with ribosomes were detected in oocytes from primary follicles (unilaminar and bilaminar) (Figure 4A), while in oocytes from secondary follicles (multilaminar) no membranous circular structures were observed, and numerous small dark vesicles distributed throughout the ooplasm (mean diameter of 100 ± 1.0 nm) were specifically labelled with the anti-PDI antibody (Figure 4B) [100]. These data agreed with observations made in growing human oocytes, which contain primary follicles in which the rough endoplasmic reticulum (RER) is sparse and consist of elongated cisternae studded with ribosomes in perinuclear disposition, decreasing in secondary and antral follicles and being absent from mature oocytes [101]. To respond to the fertilizing sperm, the egg cortex must be able to provide a full calcium response [102]. For this, the ER adopts a cisternae conformation at the beginning of oocyte growth, to be adapted to the high protein requirements and modify their morphology to provide the calcium response. The spherical shape adopted by the lamellae of the endoplasmic reticulum was reported in normal growing oocytes from some, but not all, mammalian species [103,104], and this appearance was more common in oocytes expressing significant amounts of proteins during growth after microinjection of an expression vector [105,106,107], suggesting that they form in response to an increased demand of ZP protein synthesis.

**Figure 5:**
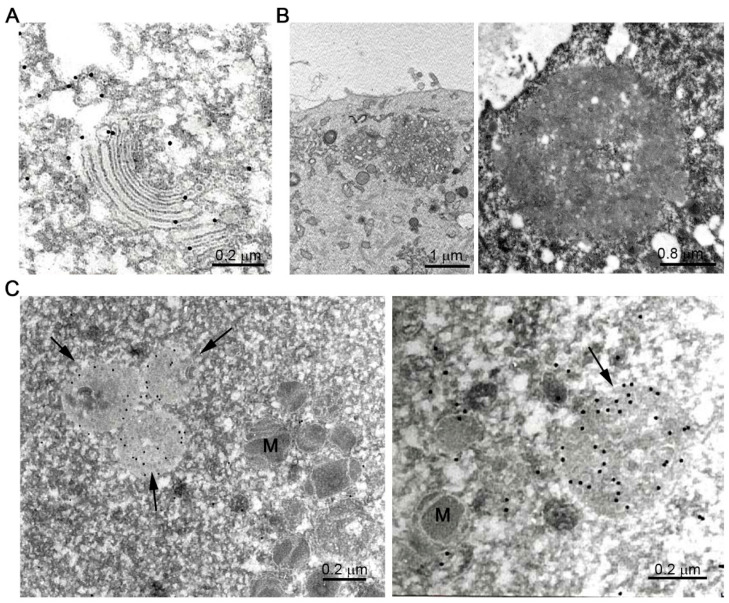
Electron microscopical analysis of oocyte organelles involved in protein trafficking. (**A**) Human prophase I oocyte immunolabeled with anti-human ZP3 antibody. The Golgi apparatus showed a moderate reactivity. (**B**) Inmatured mice oocytes. Multivesicular aggregates (MVA) consisted of multiple vesicles embedded in an amorphous material, and the majority of them were found in close proximity to the oolemma. (**C**) Human prophase I oocyte immunolabeled with anti-human ZP3 antibody. Lysosomes like structures were strongly reactive (arrows) M: Mitochondria.

Once the protein has folded, it has to be heavily and heterogeneously glycosylated at the level of the RER and Golgi complex and then routed to the plasma membrane. ZP proteins are localized at the level of the stacked Golgi membranes (Figure 5A). The Golgi apparatus becomes larger and is transformed from a few flattened sacs to large dilated cisternae in the cortex of the cell, where it is active in exporting glycoproteins to the ZP [108]. In human oocytes, Golgi complexes have circular profiles and consist of stacks of cisternae (flattened or tubular) associated with clear vesicles [101]. Final maturation is characterized by a reduction in the size of the Golgi compartment. There is a very clear polarity in the formation of the Golgi vesicles that form on the *trans* face of the Golgi stacks in the region of the oocyte. It is possible that these polarized vesicles deliver or secrete certain components of the oocyte membrane or ZP [109]. The same authors suggested that the unusual structures, defined as multivesicular aggregates (MVA) pinching off from the Golgi complexes. If this is the case, then the polar distribution of these vesicles may contribute to the initial asymmetry and polarity on the ZP [110]. However, whether the ZP is polar or not remains controversial [21,111]. The origin of the MVA has never been traced in details and remains unknown. Morphologically, MVA consist of multiple vesicles embedded in an amorphous material, and most of them are found in close proximity to the oolemma (Figure 5B). Based on the vesicle composition, these structures may resemble late-stage endosomes, but no specific markers were observed and markers for lysosomes were negative. The only organelle marked positive was syntaxin-6, a TGN/secretory vesicle marker, which suggested that the MVA structures are secretory in nature [98]. Interestingly the authors suggested that MVA may serve to sort proteins between secretory and degradative pathways. Indeed, in human oocytes, endosome-like structures were specifically labeled with antibody anti-ZP3, while MVA were never observed (Figure 5C). These observations point out the issue that the possible oocyte digestion of ZP proteins has not been studied. Vesicle composition is highly heterogeneous in shape and content, and some of them even include cortical granule-like vesicles, leading us to suggest that the MVA might act as cortex and extracellular coordinator elements in mice.

According to the relevant literature, proper trafficking into the cell and control of the process are based on two requirements—maintaining the structures of the ZP proteins in an inactive conformation and directing ZP proteins to the plasma membrane to be specifically processed. The first requirement is accomplished through the interaction of two hydrophobic pathways, the IHP and the EHP, which prevents intracellular polymerization [96,107]. Once cleaved from the transmembrane domain, the zona ectodomain retains only one of the hydrophobic patches, which might interact with other zona ectodomains to promote polymerization and formation of the extracellular ZP [96]. The second step refers to the C-terminal of the proteins, transmembrane domain, and cytoplasmic tail. The transmembrane domain permits proteins to be tethered to a transport vesicles in both leaflets of a lipid bilayer while they are trafficking through the cell, and the cytoplasmic tail acts as cytoplasmic regulator, distinguishing between ZP proteins for independent trafficking through the cell [97]. Once in the oocyte plasma membrane, the ZP proteins are cleaved by a yet unknown protein and released to form the extracellular matrix.

## 5. Are ZP Proteins the Only Proteins That Could Interact with the Sperm?

At the end of folliculogenesis, the Graafian follicles are ruptured, which allows release of the oocytes surrounded by the cumulus cells that are captured by the fimbriae of the Fallopian tubes. The oviduct is a complex organ with different regions and a very complex pattern of folds and grooves [112,113]. The lumen of this organ is covered by an epithelium formed of ciliated and non-ciliated cells that are partly responsible for the proteins contained in the oviductal fluid [114]. The gene expression of oviductal tissues was analyzed in different species by molecular techniques and proteomic analysis [115]. These studies have provided important information and pointed to differences in the gene expression profile that depend on the region of the oviduct, the phase of the cycle and even on the presence of the sperm, the oocyte, or embryos in this region [116,117,118,119,120,121]. Additionally, proteomic analyses of oviductal secretions previously carried out in different species showed that these proteins are of a dual origin: blood plasma exudates and protein secretion by the oviductal epithelium. Subsequently, the gametes and embryos are exposed to the oviductal secretions, which could be responsible for different biochemical and physiological changes. It has long been known that the biological and biochemical properties of oocytes in the Graafian follicles are different from oviductal oocytes in hamster [122,123,124,125], pig [126,127,128], cow [129], and mouse [130]. These changes in oocyte properties induced by oviductal secretions may be responsible for the poor efficiency of assisted reproductive technologies (ARTs) described in different species (reviewed in [131]). Thus, in some species a high rate of polyspermy was detected, e.g., in pig [132,133,134,135], while in others, e.g., equine [136,137,138,139,140], very low fertilization rates were reported, suggesting the existence of species-specific roles for the oviduct. Moreover, the use of oviductal fluid, epithelial cells, oviductal proteins, or exosomes was seen to improve IVF results, as reported in porcine species [141,142,143], horse [144,145], and cow [129,146].

Oviductal proteins could induce changes in the ZP that affect different aspects, such as ZP maturation and sperm-ZP binding, or prevent polyspermy. These changes might affect the ZP composition through different mechanisms: (i) by changing the carbohydrate composition or (ii) by changing the protein composition. Both these processes will be described in more detail below.

### 5.1. Changes in Carbohydrate Composition

Previous studies performed in our laboratory and others identified changes in the ZP during ovarian follicular growth [28,147,148,149,150]. Moreover, the ZP was seen to be modified after ovulation and some oviductal factors seemed to be related with differences in the fertilizability of ovarian and oviductal oocytes [122,128,130,151,152,153].

Using lectin histochemistry, it was previously reported that the lectin binding properties of the ZP are modified after ovulation. For example, in the hamster ZP, there was a significant change in the sugar moieties and distribution of glycoproteins in the ZP following ovulation [154,155]. The carbohydrate-related changes detected in the ZP after ovulation are produced by the addition of glycoproteins of oviductal origin or changes due to the effect of glycosidases present in the oviductal environment [156,157].

A redistribution of the ZP glycosidic residues was also suggested [155,158]. In one of these studies, made in rat, the lectin binding pattern observed in the ZP of follicular oocytes differed considerably from that of ovulated oocytes. Moreover, in ovulated oocytes most of the sugar residues were homogeneously distributed within the ZP, or mainly located in its inner portion, whereas in follicular oocytes, the sugar residues were mainly located at the outer regions of the ZP, adjacent to the cumulus cells, in all developmental stages.

### 5.2. Changes in Protein Composition

After expansion and disaggregation of the cumulus, the ZP becomes more accessible to the oviductal fluid, permitting its modification by different molecules. Differences in the ZP between oviductal and follicular oocytes, called zona maturation, were mentioned by a number of authors, some of whom related the differences at the ultrastructural level [159], and others identifying specific molecules in the oviductal ZP that are not present in the ovarian ZP. Study of these proteins is crucial in order to improve the efficiency of ARTs, such as IVF and embryo culture, as described in human or in porcine species [160,161]. Some of these proteins will be discussed in more detail below.

#### 5.2.1. Osteopontin

Osteopontin (OPN), the first extracellular matrix protein identified in bone tissue, by Franzén et al. [162], plays an important role in health and was described in a large number of tissues [163,164]. OPN is secreted by the oviduct in mouse [165], in which species anti-OPN antibody treatment was seen to reduce the rates of fertilization, cleavage, and blastocyst formation in vitro in a dose-dependent way. However, in *Opn* mutant mice, embryogenesis was common and the animals were fertile [166]. This protein is also present and related with oocyte in cattle [167,168,169], pig [170] and horse [144]. In cattle, immunofluorescence was shown to have an affinity for the ZP of bovine oocytes [168].

#### 5.2.2. Lactoferrin

Lactoferrin (LF) is a glycoprotein present in different human body secretions such as milk, tears, and saliva [171,172,173,174,175]. This protein has also been detected in the oviduct of mouse [173,176], rat [176], hamster [176], and human [177]. In human, studies of LF binding to the ZP showed homogenous staining. LF caused a significant dose-dependent inhibition of sperm-ZP interaction, and the effect was already significant (*p* < 0.01) even at the lowest LF concentration used [177]. The presence of this protein could regulate in vivo fertilization and it would be interesting to test its effect in other species such as pig, in which polyspermy is very high common during IVF assays.

#### 5.2.3. Oviductin

It has been more than 30 years since hamster ZP was seen to modified by OVGP1 [122] and how this protein is bound to the ZP and involved in fertilization [152,153,178]. Years later, *OVGP1* was detected in the genome of different mammals, including monotremes [179], marsupials [180], and placentals.

OVGP1 is associated with the ZP of ovarian oocytes after ovulation in several species. OVGP1 and heparin-like glycosaminoglycans (GAGs) were demonstrated to participate in the functional modification of the ZP in pig and cow, making it more resistant to enzymatic digestion and to sperm penetration, thus contributing to the control of polyspermy [181].

However, this protein is not present or has an essential role in all species. For example, only a minor fraction of the mouse OVGP1, which is recognized by the PNA lectin, is able to bind to the ZP [130] and the *Ovgp1* gene-null mouse is fertile [182]. In other cases, such as rat or megabat, *OVGP1* is a pseudogen and consequently this protein is not expressed by the oviduct [183,184]. In these species, other members of the chitinase protein family may play an important role in the absence of OVGP1.

#### 5.2.4. Lipocalin-Type Prostaglandin D Synthase

Lipocalin-type prostaglandin D synthase (L-PGDS) is a member of the family of transport proteins known as lipocalins, and has been identified as fertility-associated protein in the bull seminal plasma [185]. The biological role of L-PGDS in male reproduction is not known (Leone et al., 2002), and its role in the female tract is currently under investigation. L-PGDS was demonstrated to be associated with bovine ZP [168], and antibody against L-PGDS reacts with in vitro matured bovine oocytes and spermatozoa, resulting in increased in vitro sperm-oocyte binding and the inhibition of fertilization. These observations suggest that L-PGDS may have a role in cattle fertilization.

## 6. Concluding Remarks

This review outlines some of the progress that has been made in our understanding of the ZP during the last quarter of a century. However, many important issues related to this matrix remain unresolved or controversial. For example, what is the ZP structure like in species other than mouse, and is the sperm receptor the same in all species? Identification of the ZP protein responsible for binding to spermatozoa could be very useful in the development of fertility assays. Indeed, the development of biological models with ZP proteins would provide useful tools for exploring the molecular basis of gamete recognition in a variety of mammals and act as a diagnostic predictor of sperm function. These tools could be used to test for fertilizability and to select sperm with high fertilizing capacity both in livestock breeding centers and in human fertility clinics to improve the effectiveness of ARTs [186]. Furthermore, since ZP gene mutations are a potential cause of infertility in women, the use of animal models more similar to human than *Mus musculus* could help to understand such alterations. In this respect, CRISPR technology could serve as a valuable tool to shed light on the functionality of the different ZP proteins, as already demonstrated in rabbit species [47].

On the other hand, ZP proteins are also attractive targets for immunocontraception [187]. The newly discovered proteins described in several species should be investigated in order to develop successful vaccines for wildlife management but also for feral cats and dogs. Such vaccines would be important from biological, ecological, and economic perspectives, and more acceptable from a moral point of view than other methods commonly used to control animal populations. For instance, marsupials are of fundamental importance to the Australian and New Zealand environment, but several species have become overly abundant in some areas.

Moreover, any study of the ZP should be linked to the analysis of different oviductal proteins that interact with this matrix. These proteins could help us understand what really happens in vivo and so be used to improve ARTs. Not only would in vitro culture be enhanced but also, the conservation and transport of oocytes and embryos could be improved.

Future studies carried out in several species using CRISPR-Cas9 technology will provide valuable information about all these topics: the role played by ZP proteins in ZP formation, the ZP structure, fertilization, interaction with oviductal proteins, and embryo development.

## Data Availability

The sequences used to reconstruct the phylogeny presented in Figure S1 were retrieved from Genbank (https://www.ncbi.nlm.nih.gov/genbank/, accessed on 22 March 2021) and Ensembl (http://www.ensembl.org/index.html, accessed on 22 March 2021) following Feng et al., 2018 and Moros-Nicolas et al., 2018.

## Supplementary Materials

Supplementary materials can be found at https://www.mdpi.com/1422-0067/22/6/3276/s1.
