# Supplementary material for: New Insights into the Mammalian Egg Zona Pellucida
Source: Int J Mol Sci. 2021 Mar 23;22(6):3276. doi: 10.3390/ijms22063276 (PMC8005149; doi:10.3390/ijms22063276)
Supplement: Supplementary file 1 [file ijms-22-03276-s001.pdf]

Supplementary Materials

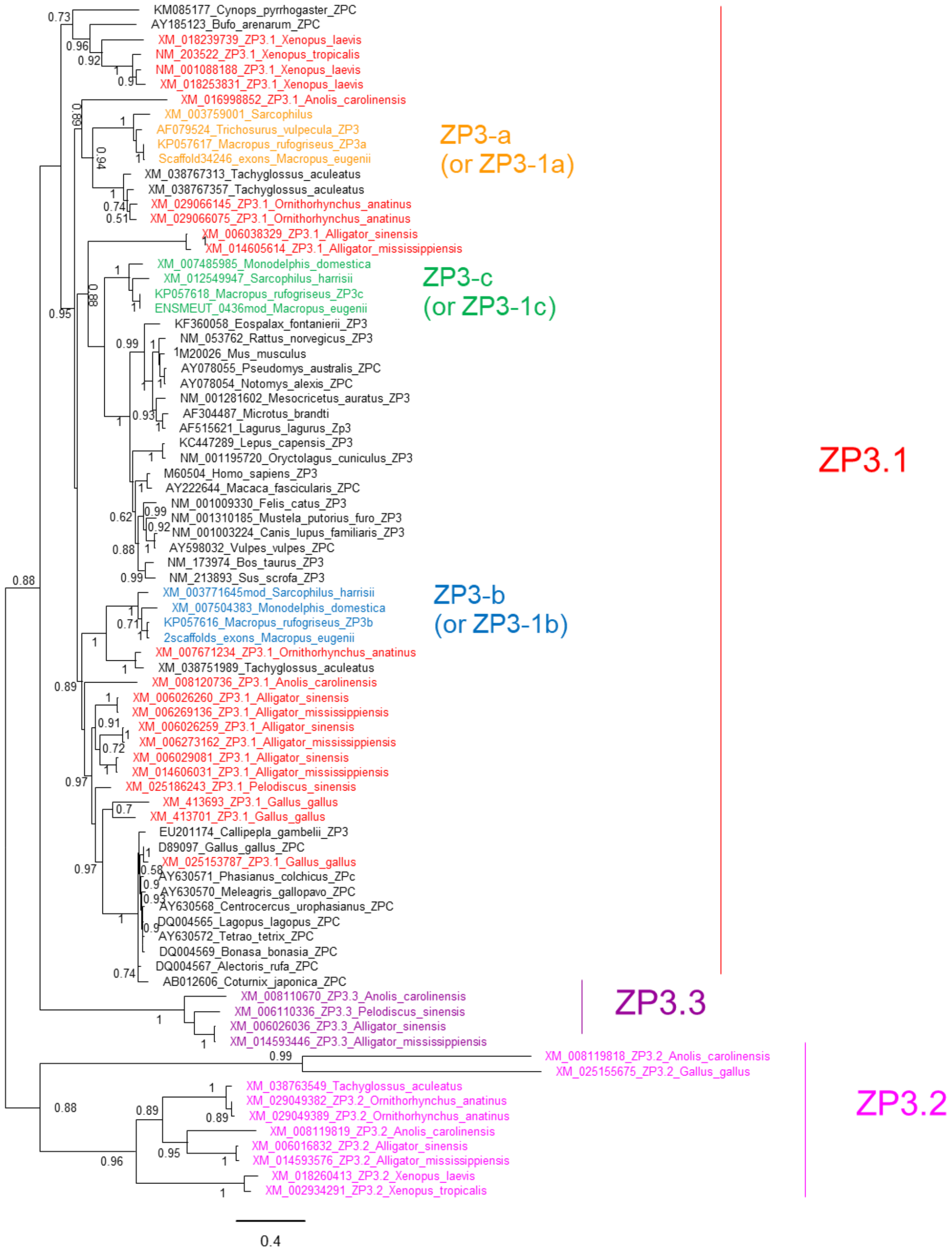

Figure S1. Phylogeny of ZP3 genes in tetrapodes reconstructed with PhyML. Sequences in red, pink and violet correspond respectively to ZP3.1, ZP2.2 and ZP3.3 of Feng et al., 2018. Sequences in orange, blue and green correspond respectively to the marsupial ZP3-a, ZP3-b and ZP3-c of Moros-Nicolas et al., 2018. The supports (aLRT values) are indicated on the nodes for values above 0.5.

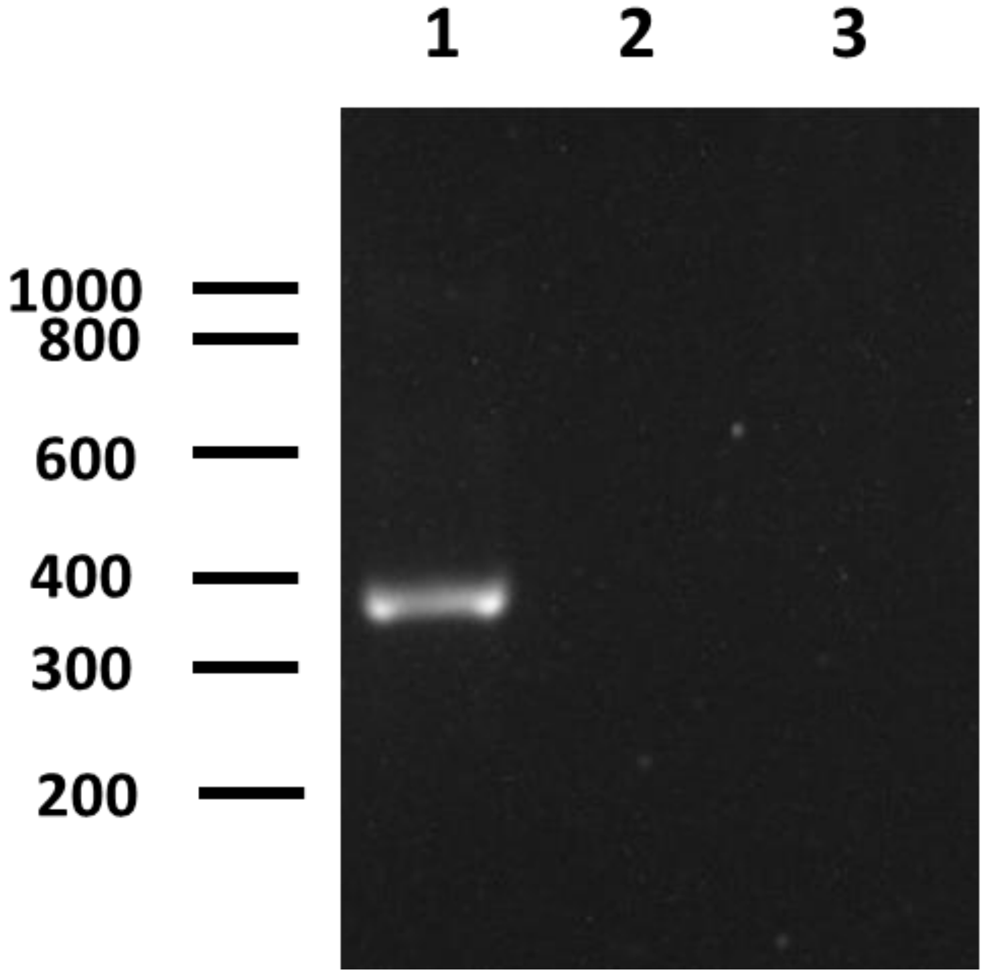

Figure S2. Analysis of *ZPAX* gene expression in Bennett's wallaby ovary as determined by reverse transcription–polymerase chain reaction (RT-PCR). In line 1 a fragment of *GAPDH* gene is shown as control. In lines 2 and 3 no fragments of *ZPAX* gene were amplified.

Table S1. Lectin histochemistry of the ZP from different mammalian species before and after different treatments.

LECTIN	Human	Mouse	Rat	Hamster	Rabbit	Hare	Cat	Dog	Pig	Cow
**Con A**	+ #<br>[1]<br><br>+ #<br>int. fail.<br>[2]<br><br>+ #<br>[3]	+ # unif.<br>[4]<br><br>± ext.<br>[5]<br><br>+ #<br>[6]	+ # unif.<br>[4]<br><br>+ # unif.<br>[11]<br><br>+ unif.<br>[12]<br><br>± ext.<br>[5]	+ # unif.<br>[4]<br><br>± ext.<br>[5]	± ext.<br>[5]<br>-<br>[16]	-<br>[16]	-<br>[5]<br><br>-<br>[17]	-<br>[5]<br><br>-<br>[17]<br><br>- primary and secondary<br>[18]<br><br>± int.<br>[19]<br><br>- primary<br>[19]	-<br>[5]<br><br>-<br>[20]<br><br>+ unif. antral<br>[21]<br><br>- primordial, primary, multilaminar and antral<br>[22]<br><br>- ^{*D}<br>[22]<br><br>- preovulatory<br>[23]<br><br>+ IVM<br>[23]	-<br>[26]<br><br>- IVM<br>[26]<br><br>+ # ext. and int.<br>[26]<br><br>-<br>[27]
**LCA**	+ #<br>[1]	±<br>[5]	- #<br>[11]<br><br>±<br>[5]	±<br>[5]	+ ext.<br>[5]<br><br>-<br>[16]	-<br>[16]	-<br>[5]<br><br>-<br>[17]	±<br>[5]<br><br>-<br>[17]	±<br>[5]	
**WGA**	+ #<br>int. and ext.<br>[1]<br><br>+ # fail.<br>[2]<br><br>+ #<br>[3]<br><br>± #^{*S}<br>[3]	+ # ext.<br>[4]<br><br>+ ext. and int.<br>[5]<br><br>+ # unif.<br>[7]<br><br>+ #^{*S} int.<br>±<br>#^{*S} ext.<br>[7]<br><br>+ E unif.<br>[7]<br><br>+ E^{*S} int.<br>±<br>E^{*S} ext.<br>[7]<br><br>+ throughout follicular development<br>[8]	+ # ext.<br>[4]<br><br>-<br>[11]<br><br>± # int. and ext.<br>[11]<br><br>+ int. and ext.<br>[12]<br><br>± unif. ^{*S}<br>[12]<br><br>+<br>[5]<br><br>+ # int.<br>+ # ext.<br>[13]<br><br>+<br>#^{*S} int.<br>±<br>#^{*S} ext.<br>[13]<br><br>+ E int.<br>+ E ext.<br>[13]<br><br>+ E^{*S} int.<br>±<br>E^{*S} ext.<br>[13]	+ # ext.<br>[4]<br><br>+ ext. and int.<br>[5]<br><br>± early primary<br>+ int. ± ext. unilaminar matured to multilaminar<br>[14]<br><br>+ ^{*S}unif.<br>[14]<br><br>+ # unif.<br>[14]<br><br>+ #^{*S}unif.<br>[14]<br><br>+ # unif.<br>[15]<br><br>+ #^{*S}unif.<br>[15]<br><br>+ E unif.<br>[15]<br><br>+ E^{*S}unif.<br>[15]	+<br>[5]<br><br>+ ext. and int.<br>[16]<br><br>+ unif. preantral<br>[16]<br><br>+ ^{*S} ext. and int.<br>[16]<br><br>+ $^{*KOH/S}$ ext. and int.<br>[16]<br><br>+ ^{*D} ext. and int.<br>[16]	+ unif.<br>[16]<br><br>+ ^{*S} unif.<br>[16]<br><br>+ $^{*KOH/S}$ unif.<br>[16]<br><br>+ ^{*D} unif. [16]	-<br>[5]<br><br>+ ext. and int.<br>[17]<br><br>+ unif. preantral<br>[17]<br><br>+ ^{*S} ext. and int.<br>[17]<br><br>+ ^{*S} unif. preantral<br>[17]	+ [5]<br><br>+ ext. and int.<br>[17]<br><br>+ unif. preantral<br>[17]<br><br>+ ^{*S} ext. and int.<br>[17]<br><br>+ ^{*S} unif. preantral<br>[17]<br><br>- primary and secondary<br>[18]<br><br>+ ext.<br>[19]<br><br>+ ^{*S} ext.<br>[19]<br><br>+ $^{*KOH/S}$ ext. [19]	+<br>[5]<br><br>+ ext. and int.<br>[20]<br><br>+ unif. antral<br>[21]<br><br>+ ^{*S} unif. antral<br>[21]<br><br>+ primordial, primary, multilaminar and antral<br>[22]<br><br>+ ^{*D}<br>[22]<br><br>+ $^{*KOH/S}$<br>preovulatory<br>[23]<br><br>+ IVM$^{*KOH/S}$<br>[23]<br><br>+<br>[24]	+ ext. and int.<br>[26]<br><br>+ IVM ext. and int.<br>[26]<br><br>+ E ext. and int.<br>[26]<br><br>+<br>[27]
**S-WGA**	+ # med.<br>[1]	+ ext.<br>[5]	+ ext.<br>[11]<br><br>+ # unif.<br>[11]<br><br>+ ext.	+ ext.<br>[5]	+ ext.<br>[5]		-<br>[5]<br><br>+ ext. and int.<br>[17]	-<br>[5]<br><br>- primary<br>[18]	-<br>[5]	

			[5]					+ secondary [18]		
**PNA**	- # [1]<br><br>+ #*^{S} [1]<br><br>- # fail. [2]<br><br>- # [3]<br><br>+ #*^{S} [3]	+ [5]<br><br>+ *^{S} [5]<br><br>+ # int. ± # ext. [7]<br><br>+ E int. ± E ext. [7]<br><br>+ # [6]	+ ext. [11]<br><br>+ # unif. [11]<br><br>+ int. ± ext. [12]<br><br>+ unif. *^{S} [12]<br><br>+ [5]<br><br>+ *^{S} [5]<br><br>+ # int. ± # ext. [13]<br><br>+ E int. ± E ext. [13]	+ ext. [5]<br><br>+ *^{S} ext. [5]<br><br>± early primary + int. ± ext. unilaminar matured to multilaminar [14]<br><br>± # unif. [14]<br><br>± # unif. [15]<br><br>± E unif. [15]	± [5]<br><br>- *^{S} [5]<br><br>+ ext. [16]<br><br>+ *^{S} unif. preantral [16]<br><br>+ *^{D} ext. [16]<br><br>+ *^{D} unif. preantral [16]<br><br>+ *$^{D/S}$ unif. [16]	- [16]<br><br>+ *^{D} unif. [16]<br><br>+ *$^{D/S}$ unif. [16]	- [5]<br><br>- *^{S} [5]<br><br>- [17]<br><br>- *^{S} [17]<br><br>- *^{S} unif. preantral [17]<br><br>+ *^{D} unif. [17]<br><br>+ *^{D} unif. preantral [17]<br><br>+ *$^{D/S}$ unif. [17]<br><br>+ *$^{D/S}$ unif. preantral [17]	- [5]<br><br>- *^{S} [5]<br><br>- [17]<br><br>- *^{S} [17]<br><br>+ *$^{D/S}$ unif. [17]<br>- [19]	- [5]<br><br>- *^{S} [5]<br><br>- [20]<br><br>- *^{S} [20]<br><br>+ *$^{KOH/S}$ unif. [20]<br><br>- *^{D} antral [25]<br><br>+ *$^{D/S}$ unif. antral [25]<br><br>- antral [21]<br><br>±*^{S} unif. antral [21]<br><br>± antral [22]<br><br>- primordial, primary and multilaminar [22]<br><br>+ *^{D} [22]<br><br>- preovulatory [23]<br><br>- IVM [23]<br><br>- [24]	- [26]<br><br>- *^{S} [26]<br><br>+ *$^{KOH/S}$ unif. [26]<br><br>- IVM [26]<br><br>- IVM*^{S} [26]<br><br>+ IVM*$^{KOH/S}$ unif. [26]<br><br>- E [26]<br><br>+ E*^{S} ext. and int. [26]<br><br>+ E*$^{KOH/S}$ ext. and int. [26]<br><br>± *^{D} ext. antral [25]<br><br>+ *$^{D/S}$ unif. antral [25]<br><br>- antral [28]
**RCA-I**	+ # [1]<br><br>+ # [3]	+ # ext. [4]<br><br>+ ext. [5]<br><br>+ # int. ± # ext. [7]<br><br>+ E int. ± E ext. [7]<br><br>+ throughout follicular development [8]	+ # ext. [4]<br><br>- [11]<br><br>+ # int. [11]<br><br>± int. + ext. [12]<br><br>+ unif. *^{S} [12]<br><br>+ ext. [5]<br><br>+ # int. ± # ext. [13]<br><br>+ E int. ± E ext.	+ # ext. [4]<br><br>+ ext [5]<br><br>± early primary + int. ± ext. unilaminar matured to multilaminar [14]<br><br>+ # int. ± # ext. [14]<br><br>+ # int. ± # ext. [15]<br><br>+ E int. ± E ext. [15]	+ ext. [5]<br><br>+ [16]<br><br>+ *$^{D/S}$ unif. [16]	- [16]	+ ext. [5]<br><br>- [17]<br><br>+ *^{S} int. [17]<br><br>+ *^{S} unif. preantral [17]<br><br>+ *$^{KOH/S}$ unif. [17]<br><br>+ *^{D} unif. [17]	- [5]<br><br>- [17]<br><br>+ *^{S} ext. and int. [17]<br><br>+ *^{S} unif. preantral [17]<br><br>± unif. [19]<br><br>+ primary [19]<br><br>+ *^{S} int. [19]<br><br>+ *^{D} ext. and int. [17]	+ ext. [5]<br><br>+ int. [20]<br><br>+ *^{S} unif. [20]<br><br>+ *$^{KOH/S}$ unif. [20]<br><br>- *^{D} antral [25]<br><br>+ *$^{D/S}$ unif. antral [25]<br><br>+ int. antral [21]<br><br>+*^{S} int. and ext. antral	- [26]<br><br>- *^{S} [26]<br><br>+ *$^{KOH/S}$ unif. [26]<br><br>- IVM [26]<br><br>- IVM*^{S} [26]<br><br>+ IVM*$^{KOH/S}$ unif. [26]<br><br>- E [26]<br><br>- E*^{S} [26]

			[13]					+ *^{D} unif.<br>preantral<br>[17]<br><br>+ *$^{D/S}$ ext. and<br>int.<br>[17]<br><br>+ *$^{D/S}$ unif.<br>preantral<br>[17]	[21]<br><br>+ primordial,<br>primary,<br>multilaminar<br>and antral<br>[22]<br><br>+ *^{D}<br>[22]<br><br>+ preovulatory<br>[23]<br><br>+ IVM<br>[23]<br><br>+<br>[24]	+ E*$^{KOH/S}$ unif.<br>[26]<br><br>+ *^{D} ext. antral<br>[25]<br><br>+ *$^{D/S}$ unif.<br>antral<br>[25]
**GS-I**	- #<br>[1]<br><br>- # fail.<br>[2]	+ int.<br>[5]	+ # unif.<br>[11]<br><br>+<br>[5]	-<br>[5]	-<br>[5]		-<br>[5]	-<br>[5]	-<br>[5]	+<br>[27]
**GS-II**	- # fail.<br>[2]									
**SBA**	- #<br>[1]<br><br>- # fail.<br>[2]<br><br>- #<br>[3]<br><br>+ #*^{S}<br>[3]	+<br>[5]<br><br>+ unif.<br>throughout<br>follicular<br>development<br>[9]<br><br>+ #<br>[9]	-<br>[11]<br><br>+ # unif.<br>[11]<br><br>± int. +<br>ext.<br>[12]<br><br>+ unif. *^{S}<br>[12]<br><br>+<br>[5]	+<br>[5]	-<br>[5]<br><br>+ ext.<br>[16]<br><br>+ *^{S} ext.<br>[16]<br><br>+ *^{S} unif.<br>preantral<br>[16]<br><br>+ *$^{KOH/S}$ unif.<br>[16]<br><br>+ *^{D} ext. and<br>int.<br>[16]<br><br>+ *^{D} unif.<br>preantral<br>[16]<br><br>+ *$^{D/S}$ unif.<br>[16]	-<br>[16]<br><br>+ *^{S} unif.<br>[16]<br><br>+ *^{D} unif.<br>[16]<br><br>+ *$^{D/S}$ unif.<br>[16]	-<br>[5]<br><br>-<br>[17]<br><br>+ *^{S} unif.<br>[17]<br><br>+ *^{D} ext and<br>int.<br>[17]<br><br>+ *^{D} unif.<br>preantral<br>[17]<br><br>+ *$^{D/S}$ unif. [17]	-<br>[5]<br><br>-<br>[17]<br><br>+ *^{S} ext. and<br>int.<br>[17]<br><br>+ *^{S} unif.<br>preantral<br>[17]<br><br>-<br>[19]<br><br>+ *^{S} ext. and<br>int.<br>[19]<br><br>+ *$^{KOH/S}$ ext. and<br>int.<br>[19]<br><br>+ *^{D} ext. and<br>int.<br>[17]<br><br>+ *^{D} unif.<br>preantral<br>[17]<br><br>+ *$^{D/S}$ ext. and<br>int.<br>[17]<br><br>+ *$^{D/S}$ unif.<br>preantral<br>[17]	-<br>[5]<br><br>-<br>[20]<br><br>+ *^{S} unif.<br>[20]<br><br>+ *$^{KOH/S}$ unif.<br>[20]<br><br>- *^{D} ext. antral<br>[25]<br><br>+ *$^{D/S}$ unif.<br>antral<br>[25]<br><br>+ int. and med.<br>antral<br>[21]<br><br>- multilaminar<br>[22]<br><br>± antral<br>[22]<br><br>+ primordial<br>and primary<br>[22]<br><br>+ *^{D}<br>[22]<br><br>+ preovulatory<br>[23]<br><br>+ IVM<br>[23]<br><br>-<br>[24]	-<br>[26]<br><br>+ *^{S} ext. and<br>int.<br>[26]<br><br>+ *$^{KOH/S}$ unif.<br>[26]<br><br>- IVM<br>[26]<br><br>+ IVM*^{S} ext.<br>and int.<br>[26]<br><br>+ IVM*$^{KOH/S}$<br>unif.<br>[26]<br><br>+ E ext.<br>[26]<br><br>+ E*^{S} ext. and<br>int.<br>[26]<br><br>+ E*$^{KOH/S}$ unif.<br>[26]<br><br>+ *^{D} ext. antral<br>[25]<br><br>+ *$^{D/S}$ unif.<br>antral<br>[25]<br><br>± antral<br>[28]<br><br>+ *^{S} antral [28]
**DBA**	- #	+ int.	-	-	-	-	-	-	-	-

	[1]<br><br>- # fail.<br>[2]<br><br>- #<br>[3]<br><br>- #^{*S}<br>[3]	[5]<br><br>+ unif. throughout follicular development<br>[9]<br><br>+ #<br>[9]	[11]<br><br>+ # unif.<br>[11]<br><br>-<br>[5]<br><br>- #<br>[13]<br><br>- E<br>[13]	[5]<br><br>-<br>[14]<br><br>± # unif.<br>[14]<br><br>± # unif.<br>[15]<br><br>± E unif.<br>[15]	[5]<br><br>-<br>[16]	[16]	[5]<br><br>-<br>[17]	[5]<br><br>-<br>[17]<br><br>-<br>[19]	[5]<br><br>-<br>[20]<br><br>- ^{*S}<br>[20]<br><br>- $^{*KOH/S}$<br>[20]<br><br>- $^{*D/S}$ antral<br>[25]<br><br>- int. antral<br>[21]<br><br>- primordial, primary, multilaminar and antral<br>[22]<br><br>- ^{*D}<br>[22]<br><br>- preovulatory<br>[23]<br><br>- IVM<br>[23]<br><br>-<br>[24]	[26]<br><br>- ^{*S}<br>[26]<br><br>- $^{*KOH/S}$<br>[26]<br><br>- IVM<br>[26]<br><br>- IVM^{*S}<br>[26]<br><br>- IVM$^{*KOH/S}$<br>[26]<br><br>- E<br>[26]<br><br>- E^{*S}<br>[26]<br><br>+ E$^{*KOH/S}$<br>[26]<br><br>- $^{*D/S}$ antral<br>[25]<br><br>- antral<br>[28]
**UEA-I**	- #<br>[1]<br><br>- # fail.<br>[2]<br><br>- #<br>[3]<br><br>- #^{*S}<br>[3]	-<br>[5]<br><br>-<br>[8]<br><br>-<br>[10]<br><br>- #<br>[10]	-<br>[11]<br><br>± #<br>[11]<br><br>-<br>[5]<br><br>- #<br>[13]<br><br>- E<br>[13]<br><br>-<br>[10]<br><br>- #<br>[10]	-<br>[5]<br><br>-<br>[10]<br><br>- #<br>[10]<br><br>-<br>[14]<br><br>- #<br>[14]<br><br>- #<br>[15]	-<br>[5]<br><br>-<br>[16]	-<br>[16]	-<br>[5]<br><br>-<br>[17]	-<br>[5]<br><br>-<br>[17]<br><br>± int.<br>[19]<br><br>- primary<br>[19]<br><br>- primary and secondary [18]	-<br>[5]<br><br>-<br>[20]<br><br>± int. antral<br>[21]<br><br>-primordial and primary<br>[22]<br><br>+ multilaminar and antral [22]<br><br>+ ^{*D}<br>[22]<br><br>- preovulatory<br>[23]<br><br>- IVM<br>[23]<br><br>+/-<br>[24]	-<br>[26]<br><br>- IVM<br>[26]<br><br>- E<br>[26]<br><br>-<br>[27]
**AAA**	+ #<br>[3]	+ # int.<br>± # ext.<br>[7]<br><br>+ E int.<br>± E ext.<br>[7]<br><br>+ throughout follicular development<br>[8]<br><br>+	+ unif.<br>[12]<br><br>+ # int.<br>± # ext.<br>[13]<br><br>± E unif.<br>[13]<br><br>+<br>[10]<br><br>+ $^{*\alpha\text{-gal.}}$	+<br>[10]<br><br>+ $^{*\alpha\text{-gal.}}$<br>[10]<br><br>+ #<br>[10]<br><br>+ $^{*\alpha\text{-gal.}}$ #<br>[10]					- primordial, primary, multilaminar and antral<br>[22]<br><br>+ ^{*D}<br>[22]	

		[10]<br><br>+ *$^{\alpha\text{-gal.}}$<br>[10]<br><br>+ #<br>[10]<br><br>+ *$^{\alpha\text{-gal.}}$ #<br>[10]	[10]<br><br>+ #<br>[10]<br><br>+ *$^{\alpha\text{-gal.}}$ #<br>[10]	+ throughout follicular development<br>[14]<br><br>+ #<br>[14]<br><br>+ # unif.<br>[15]<br><br>+ E int.<br>± E ext.<br>[15]						
**DSA**	+ #<br>[3]	+ # ext.<br>± # int.<br>[7]<br><br>+ E int.<br>± E ext.<br>[7]<br><br>+ throughout follicular development<br>[8]	+ unif.<br>[12]<br><br>+ # int.<br>± # ext.<br>[13]<br><br>+ E int.<br>± E ext.<br>[13]	± early primary<br>+ int. ± ext. unilaminar matured to multilaminar<br>[14]<br><br>+ # int.<br>± # ext.<br>[14]<br><br>+ # int.<br>± # ext.<br>[15]<br><br>+ E int.<br>± E ext.<br>[15]						
**LFA**	+ #<br>[3]<br><br>- #*^{S}<br>[3]	+ # int.<br>± # ext.<br>[7]<br><br>+ E int.<br>± E ext.<br>[7]<br><br>+ throughout follicular development<br>[8]<br><br>- *^{S}<br>[8]	+ unif.<br>[12]<br><br>+ # int.<br>± # ext.<br>[13]<br><br>+ E int.<br>± E ext.<br>[13]						- primordial and primary<br>[22]<br><br>+ multilaminar and antral<br>[22]<br><br>- *^{D} primordial and primary<br>[22]<br><br>+ *^{D} multilaminar and antral<br>[22]	+ antral unif.<br>[28]<br><br>- *^{S} antral [28]
**MAA**	+ #<br>[3]<br><br>- #*^{S}<br>[3]	+ # int.<br>± # ext.<br>[7]<br><br>+ E int.<br>± E ext.<br>[7]<br><br>+ throughout follicular development<br>[8]<br><br>- *^{S}<br>[8]	+ unif.<br>[12]<br><br>+ # int.<br>± # ext.<br>[13]<br><br>+ E int.<br>± E ext.<br>[13]	± early primary<br>+ int. ± ext. unilaminar matured to multilaminar<br>[14]<br><br>+ # unif.<br>[14]<br><br>- #*^{S}<br>[14]<br><br>+ # unif.<br>[15]<br><br>- #*^{S}<br>[15]<br><br>+ E unif.<br>[15]					-primordial<br>[22]<br><br>± primary<br>[22]<br><br>+ multilaminar and antral<br>[22]<br><br>- *^{D} primordial<br>[22]<br><br>± *^{D} primary<br>[22]<br><br>+ *^{D} multilaminar and antral<br>[22]	+ antral unif.<br>[28]<br><br>- *^{S} antral [28]
**PHA-E**	+ #<br>[3]									
**PHA-L**	+ #	+ #								

	[3]	[6]								
**BSA-I-B4**	- #<br>[3]	± # int.<br>- # ext.<br>[7]<br><br>± E int.<br>- E ext.<br>[7]		-<br>[14]<br><br>± # unif.<br>[14]<br><br>+ unif.<br>[15]<br><br>+ E int.<br>± E ext.<br>[15]						
**GNA**	- #<br>[3]	-<br>[8]	- #<br>[13]<br><br>- E<br>[13]	-<br>[14]<br><br>- #<br>[14]<br><br>- #<br>[15]					- primordial, primary, multilaminar and antral<br>[22]<br><br>- ^{*D} primordial, primary, multilaminar and antral<br>[22]	
**HPA**	- #<br>[3]<br><br>- #^{*S}<br>[3]	+ # int.<br>- # ext.<br>[7]<br><br>+ E int.<br>- E ext.<br>[7]<br><br>+ int. primary to preovulatory<br>[8]<br><br>+ ^{*S} throughout follicular development<br>[8]	- #<br>[13]<br><br>- E<br>[13]	-<br>[14]<br><br>± ^{*S}<br>[14]<br><br>+ #<br>[14]<br><br>+ # unif.<br>[15]<br><br>+ E int.<br>± E ext.<br>[15]					- primordial, primary, multilaminar and antral [22]<br><br>- ^{*D}<br>[22]<br><br>- preovulatory<br>[23]<br><br>- IVM<br>[23]	- antral<br>[28]
**LTA**	- #<br>[3]<br><br>+ #^{*S}<br>[3]	-<br>[8]<br><br>-<br>[10]<br><br>- #<br>[10]	- #<br>[13]<br><br>- E<br>[13]<br><br>-<br>[10]<br><br>- #<br>[10]	-<br>[10]<br><br>- #<br>[10]<br><br>-<br>[14]<br><br>- #<br>[14]<br><br>- #<br>[15]	-<br>[16]	-<br>[16]	-<br>[17]	-<br>[17]<br><br>-<br>[19]	-<br>[20]<br><br>- int. antral<br>[21]<br><br>- primordial, primary, multilaminar and antral [22]<br><br>- ^{*D}<br>[22]<br><br>- preovulatory<br>[23]<br><br>- IVM<br>[23]	-<br>[26]<br><br>- IVM<br>[26]<br><br>- E<br>[26]
**MAL II**									+ preovulatory<br>[23]<br><br>- $^{*KOH/S}$ preovulatory<br>[23]<br><br>+ IVM<br>[23]<br><br>- $^{*KOH/S}$ IVM<br>[23]	

**SNA**	- # [3]	- [8]	- # [13]<br><br>- E [13]	- [14]<br><br>- # [14]<br><br>- # [15]					- primordial, primary, multilaminar and antral [22]<br><br>- *^{D} primordial and primary [22]<br><br>+ *^{D} multilaminar and antral [22]<br><br>- preovulatory [23]<br><br>- *$^{KOH/S}$ preovulatory [23]<br><br>+ IVM ext. and int. [23]<br><br>- *$^{KOH/S}$ IVM [23]	+ antral unif. [28]<br><br>- *^{S} antral [28]
**STA**	- # [3]									
**GSA-II**					- [16]	- [16]	- [17]	- [17]	- [20]<br><br>- *^{D} antral [25]<br><br>± *$^{D/S}$ unif. antral [25]<br><br>- preovulatory [23]<br><br>- IVM [23]	- [26]<br><br>- IVM [26]<br><br>- E [26]<br><br>- *^{D} antral [25]<br><br>+ *$^{D/S}$ unif. antral [25]
**GSA-IB4**					- [16]	- [16]	- [17]<br><br>+ *^{D} unif. [17]<br>+ *$^{D/S}$ unif. [17]	- [17]<br><br>+ *$^{D/S}$ unif. [17]	± *^{D} ext. antral [25]<br><br>+ *$^{D/S}$ unif. antral [25]	+ *^{D} ext. antral [25]<br><br>+ *$^{D/S}$ unif. antral [25]
**BPA**	- # fail. [2]									
**MPA**	+ # fail. [2]<br><br>+ # [3]									
**PSA**	+ # int. fail. [2]							- primary [18]<br><br>+ secondary [18]		
**Jacalin**	+ # [3]									
**ECL**								- primary [18]<br><br>+ secondary [18]		
**GSL-I**								- primary and secondary [18]		

**GSL-II**								± primary [18]<br><br>+ secondary [18]		
**LPA**										+<br>[27]
**LEL**								± primary [18]<br><br>+ secondary [18]		
**DSL**								- primary and secondary [18]		
**SJA**								- primary and secondary [18]		
**PVL**								- primary and secondary [18]		
**PVE**								- primary and secondary [18]		
**VVA**								± primary and secondary [18]		

Positive binding: **+**; Weakly positive binding: **±** (sometimes used for showing intensity differences between the external and internal ZP surface**)**; Negative binding: -
Binding localized in the external ZP surface: **ext.**; internal ZP surface: **int.**; medium ZP: **med.** or uniform binding: **unif.**
Most analysis were carried out using sections of ovaries. Some of them used isolated tubal unfertilized oocytes: **#;** fertilized eggs: **E** or *in vitro* matured oocytes: **IVM.** Oocytes failing to fertilize in vitro: **fail.**
*****: lectin binding procedures were preceded by different pre-treatments. Sialidase digestion: **S**; Saponification/sialidase digestion: **KOH/S**; Desulphation process: **D;** Desulphation/sialidase digestion: **D/S;** α-galactosidase: **α-gal.**
Some references distinguish between **primordial**, **primary**, **secondary**, **multilaminar** and **antral** follicles. Some lectins bound the ZP of ovarian oocytes starting from the early stages of follicular growth (primary follicles) to the Graafian follicle stage. That is designated as: **Throughout follicular development.**

References

1. Maymon, B. B. S.; Maymon, R.; Ben-Nun, I.; Ghetler, Y.; Shalgi, R.; & Skutelsky, E. (1994). Distribution of carbohydrates in the zona pellucida of human oocytes. *J. Reprod. Fertil.* ***1994***, 102, 81–86, doi:10.1530/jrf.0.1020081.
2. Talevi, R.; Gualtieri, R.; Tartaglione, G.; Fortunato, A. Heterogeneity of the zona pellucida carbohydrate distribution in human oocytes failing to fertilize in vitro. *Hum. Reprod.* **1997**, *12*, 2773–2780, doi:10.1093/humrep/12.12.2773.
3. Jiménez-Movilla, M.; Avilés, M.; Gómez-Torres, M.J.; Fernández-Colom, P.J.; Castells, M.T.; de Juan, J.; Romeu, A.; Ballesta, J. Carbohydrate analysis of the zona pellucida and cortical granules of human oocytes by means of ultrastructural cytochemistry. *Hum. Reprod.* **2004**, *19*, 1842–1855, doi:10.1093/humrep/deh311.
4. Nicolson, G.L.; Yanagimachi, R.; Yanagimachi, H. Ultrastructural localization of lectin-binding sites on the zonae pellucidae and plasma membranes of mammalian eggs. *J. Cell Biol.* **1975**, *66*, 263–274, doi:10.1083/jcb.66.2.263.
5. Skutelsky, E.; Ranen, E.; Shalgi, R. Variations in the distribution of sugar residues in the zona pellucida as possible species-specific determinants of mammalian oocytes. *J. Reprod. Fertil.* **1994**, *100*, 35–41, doi:10.1530/jrf.0.1000035.
6. Williams, S.A.; Xia, L.; Cummings, R.D.; McEver, R.P.; Stanley, P. Fertilization in mouse does not require terminal galactose or N-acetylglucosamine on the zona pellucida glycans. *J. Cell Sci.* **2007**, *120*, 1341–1349, doi:10.1242/jcs.004291.

7. Avilés, M.; Jaber, L.; Castells, M.T.; Ballesta, J.; Kan, F.W.K. Modifications of carbohydrate residues and ZP2 and ZP3 glycoproteins in the mouse zona pellucida after fertilization. *Biol. Reprod.* **1997**, *57*, 1155–1163, doi:10.1095/biolreprod57.5.1155.
8. Avilés, M.; El-Mestrgah, M.; Jaber, L.; Castells, M.T.; Ballesta, J.; Kan, F.W.K. Cytochemical demonstration of modification of carbohydrates in the mouse zona pellucida during folliculogenesis. *Histochem. Cell Biol.* **2000**, *113*, 207–219, doi:10.1007/s004180050440.
9. Avilés, M.; Castells, M.T.; Abascal, I.; Martínez-Menárguez, J.A.; Dráber, P.; Kan, F.W.K.; Ballesta, J. Cytochemical localization of GalNAc and GalNAcβ1,4Galβ1,4 disaccharide in mouse zona pellucida. *Cell Tissue Res.* **1999**, *295*, 269–277, doi:10.1007/s004410051233.
10. Avilés, M.; Okinaga, T.; Shur, B.; Ballesta, J. Differential expression of glycoside residues in the mammalian zona pellucida. *Mol. Reprod. Dev.* **2000**, *57*, 296–308, doi:10.1002/1098-2795(200011)57:3<296::AID-MRD12>3.0.CO;2-R.
11. Shalgi, R.; Maymon, R.; Bar-Shira (Maymon), B.; Amihai, D.; Skutelsky, E. Distribution of lectin receptors sites in the zona pellucida of follicular and ovulated rat oocytes. *Mol. Reprod. Dev.* **1991**, *29*, 365–372, doi:10.1002/mrd.1080290408.
12. Avilés, M.; Martínez-Menárguez, J.A.; Castells, M.T.; Madrid, J.F.; Ballesta, J. Cytochemical characterization of oligosaccharide side chains of the glycoproteins of rat zona pellucida: An ultrastructural study. *Anat. Rec.* **1994**, *239*, 137–149, doi:10.1002/ar.1092390204.
13. Avilés, M.; Jaber, L.; Castells, M.T.; Kan, F.K.W.; Ballesta, J. Modifications of the lectin binding pattern in the rat zona pellucida after in vivo fertilization. *Mol. Reprod. Dev.* **1996**, *44*, 370–381, doi:10.1002/(SICI)1098-2795(199607)44:3<370::AID-MRD11>3.0.CO;2-4.
14. El-Mestrah, M.; Kan, F.W.K. Distribution of lectin-binding glycosidic residues in the hamster follicular oocytes and their modifications in the zona pellucida after ovulation. *Mol. Reprod. Dev.* **2001**, *60*, 517–534, doi:10.1002/mrd.1117.
15. El-Mestrah, M.; Kan, F.W.K. Variations in modifications of sugar residues in hamster zona pellucida after in vivo fertilization and in vitro egg activation. *Reproduction* **2002**, *123*, 671–682, doi:10.1530/rep.0.1230671.
16. Parillo, F.; Verini-Supplizi, A. Glycohistochemistry of the zona pellucida of developing oocytes in the rabbit and hare. *Res. Vet. Sci.* **2001**, *70*, 257–264, doi:10.1053/rvsc.2001.0472.
17. Parillo, F.; Verini-Supplizi, A. Glycohistochemical investigation of canine and feline zonae pellucidae of preantral and antral oocytes. *Acta Histochem.* **1999**, *101*, 127–146, doi:10.1016/S0065-1281(99)80013-0.
18. Blackmore, D.G.; Baillie, L.R.; Holt, J.E.; Dierkx, L.; Aitken, R.J.; McLaughlin, E.A. Biosynthesis of the canine zona pellucida requires the integrated participation of both oocytes and granulosa cells. *Biol. Reprod.* **2004**, *71*, 661–668, doi:10.1095/biolreprod.104.028779.
19. Parillo, F.; Zelli, R.; Verini-Supplizi, A.; Fagioli, O.; Gargiulo, A.M. Topographical localisation of glucidic residues and their variations in the canine zona pellucida during folliculogenesis. *J. Mol. Histol.* **2005**, *36*, 131–137, doi:10.1007/s10735-004-5820-4.
20. Parillo, F.; Stradaioli, G.; Dall'Aglio, C.; Verini-Supplizi, A. Characterization of the complex carbohydrates in the zona pellucida of mammalian oocytes using lectin histochemistry. *Vet. Res. Commun.* **1996**, *20*, 225–236, doi:10.1007/BF00366920.
21. Parillo, F.; Dall'Aglio, C.; Verini Supplizi, A.; Ceccarelli, P.; Gargiulo, A. Immunogold study on lectin binding in the porcine zona pellucida and granulosa cells - PubMed. *Eur J Histochem* **2003**, *47*, 353–358.

22. Pastor, L.M.; Lucas, X.; Pallares, J.; Bernal-Mañas, C.M.; Martinez, E.A.; Roca, J.; Vazquez, J.M.; Morales, E.; Beltran, E.; Zuasti, A.; et al. Characterization of glycoside residues of porcine zona pellucida and ooplasm during follicular development and atresia. *Mol. Reprod. Dev.* **2008**, *75*, 1473–1483, doi:10.1002/mrd.20886.

23. Accogli, G.; Douet, C.; Ambruosi, B.; Martino, N.A.; Uranio, M.F.; Deleuze, S.; Dell'Aquila, M.E.; Desantis, S.; Goudet, G. Differential expression and localization of glycosidic residues in in vitro- and in vivo-matured cumulus-oocyte complexes in equine and porcine species. *Mol. Reprod. Dev.* **2014**, *81*, 1115–1135, doi:10.1002/mrd.22432.

24. Mendes, M. H.; Pinto, M. H.; Gimeno, E. J.; Barbeito, C. G.; de Sant'Ana, F. J. Lectin histochemical pattern on the normal and cystic ovaries of sows. *Reprod. Domest. Anim*. **2019**, 54, 1366-1374. doi: 10.1111/rda.13531

25. Parillo, F.; Fagioli, O.; Dall'Aglio, C.; Verini-Supplizi, A. Lectin histochemical detection of sulfoglycans in the zona pellucida of mammalian antral oocytes. *Acta histochem*. **2000**, 102, 193-202. doi: 10.1078/S0065-1281(04)70028-8

26. Verini-Supplizi, A.; Monaci, M.; Stradaioli, G.; Greve, T.; Parillo, F. Identification of glycoconjugates in the zona pellucida of in vitro matured and tubal unfertilized bovine oocytes by lectin histochemistry. *Anim. Reprod. Sci*. **1996**, 43, 99-111.

27. De Paz, P.; Sanchez, A. J.; De la Fuente, J.; Chamorro, C. A.; Alvarez, M.; Anel, E.; Anel, L. Ultrastructural and cytochemical comparison between calf and cow oocytes. *Theriogenology*. **2001**, *55*, 1107-1116. doi: 10.1016/s0093-691x(01)00470-8

28. Velásquez, J. G.; Canovas, S.; Barajas, P.; Marcos, J.; Jiménez-Movilla, M.; Gallego, R. G.; ... & Coy, P. Role of sialic acid in bovine sperm–zona pellucida binding. *Mol. Reprod. Dev.* **2007**, *74*, 617-628, doi: 10.1002/mrd.20619.

Table S2. Lectin histochemistry of the ZP from different mammalian and marsupial species before and after different treatments.

LECTIN	Wild boar	Fallow deer	Red deer	Roe deer	Buffalo	Fat-tailed dunnart	Southern brown bandicoot	Grey short-tailed opossum	Brushtail possum	Ringtail possum	Koala	Eastern grey kangaroo
**Con A**	- [1]<br>- *S [1]<br>- *KOH/S [1]<br>- *D [1]	- [2]<br>- *S [2]<br>- *KOH/S [2]<br>- *D [2]	- [2]<br>- *S [2]<br>- *KOH/S [2]<br>- *D [2]	- [2]<br>- *S [2]<br>- *KOH/S [2]<br>- *D [2]	- [3]<br>- *S [3]	- [4]	- [4]	- [4]	- [4]	- [4]	- [4]	- [4]
**LCA**	- [1]<br>- * S [1]<br>- *KOH/S [1]<br>- *D [1]				- [3]<br>- * S [3]<br>- *KOH/S [3]<br>- *D [3]							
**WGA**	+ ext. and int. [1]<br>+ *S ext. and int. [1]<br>+ *KOH/S ext and int. [1]	+ ext. and int. [2]<br>+ *S ext. and int. [2]<br>+ *KOH/S ext and int. [2]	+ ext. and int. [2]<br>+ unif. preantral [2]<br>+ *S ext. and int. [2]<br>+ *KOH/S ext. and int. [2]	+ ext. and int. [2]<br>+ unif. preantral [2]<br>+ *S ext. and int. [2]<br>+ *KOH/S ext. and int. [2]	+ ext. and int. [3]<br>+ *S [3]<br>+ *KOH/S [3]	+ [4]<br>+ *S [4]	+ [4]<br>+ *S [4]	+ [4]<br>+ *S [4]	+ [4]<br>+ *S [4]	+ [4]<br>+ *S [4]	+ [4]<br>+ *S [4]	+ [4]<br>+ *S [4]
**PNA**	- [1]<br>+ *KOH/S [1]<br>+ *D ext. [1]<br>+ *D unif. preantral [1]<br>+ *D/S [1]	- [2]<br>+ *S ext and int. [2]<br>+ *S unif. preantral [2]<br>+ *KOH/S unif. [2]<br>+ *D ext. [2]<br>+ *D unif. preantral [2]	- [2]<br>+ *S ext. and int. [2]<br>+ *S unif. preantral [2]<br>+ *KOH/S ext. and int. [2]<br>+ *KOH/S unif. preantral [2]<br>+ *D ext. and int.	- [2]<br>+ *S unif. [2]<br>+ *S unif. preantral [2]<br>+ *KOH/S unif. [2]<br>+ *D unif. [2]	- [3]<br>- *S [3]<br>+ *KOH/S [3]	- [4]<br>+ *S [4]	+ [4]<br>+ *S [4]	+ [4]<br>+ *S [4]	+ [4]<br>+ *S [4]	+ [4]<br>+ *S [4]	+ [4]<br>+ *S [4]	+ [4]<br>+ *S [4]

			[2]<br>+ *D unif. preantral<br>[2]									
**RCA-I**	-<br>[1]<br>+ *D/S<br>[1]	-<br>[2]<br>+ *S unif.<br>[2]<br>+ *KOH/S<br>[2]<br>+ *D<br>[2]	-<br>[2]<br>+ *S unif.<br>[2]<br>+ *KOH/S<br>[2]<br>+ *D<br>[2]	-<br>[2]<br>+ *S unif.<br>[2]<br>+ *KOH/S [2]<br>+ *D<br>[2]	-<br>[3]<br>+ *S<br>[3]<br>+ *KOH/S<br>[3]							
**RCA-II**						+<br>[4]<br>+ *S<br>[4]	+<br>[4]<br>+ *S<br>[4]	-<br>[4]<br>- *S<br>[4]	-<br>[4]<br>- *S<br>[4]	+<br>[4]<br>+ *S<br>[4]	+<br>[4]<br>+ *S<br>[4]	-<br>[4]<br>+ *S<br>[4]
**SBA**	-<br>[1]<br>+ *S<br>[1]<br>+ *KOH/S<br>[1]<br>+ *D<br>[1]<br>+ *D/S<br>[1]	-<br>[2]<br>+ *S unif.<br>[2]<br>+ *KOH/S<br>unif.<br>[2]<br>+ *D<br>[2]	-<br>[2]<br>+ *S int.<br>[2]<br>+ *S unif.<br>preantral<br>[2]<br>+ *KOH/S int.<br>[2]<br>+ *KOH/S<br>unif.<br>preantral<br>[2]<br>+ *D ext.<br>and int.<br>[2]<br>+ *D unif.<br>preantral<br>[2]	-<br>[2]<br>+ *S unif.<br>[2]<br>+ *KOH/S<br>unif.<br>[2]<br>+ *D ext.<br>and int.<br>[2]<br>- *D<br>preantral<br>[2]	-<br>[3]<br>+ *S<br>[3]<br>+ *KOH/S<br>[3]	-<br>[4]<br>+ *S<br>[4]	+<br>[4]<br>+ *S<br>[4]	-<br>[4]<br>- *S<br>[4]	-<br>[4]<br>+ *S<br>[4]	-<br>[4]<br>+ *S<br>[4]	-<br>[4]<br>+ *S<br>[4]	-<br>[4]<br>+ *S<br>[4]
**DBA**	-<br>[1]<br>- *S<br>[1]<br>- *KOH/S<br>[1]<br>- *D<br>[1]	-<br>[2]<br>- *S<br>[2]<br>- *KOH/S<br>[2]<br>- *D<br>[2]	-<br>[2]<br>- *S<br>[2]<br>- *KOH/S<br>[2]<br>- *D<br>[2]	-<br>[2]<br>- *S<br>[2]<br>- *KOH/S<br>[2]<br>- *D<br>[2]	- *KOH/S<br>[3]<br>- *D<br>[3]							
**UEA-I**	-<br>[1]<br>- *S	-<br>[2]<br>- *S	-<br>[2]<br>- *S	-<br>[2]<br>- *S	-<br>[3]<br>-*S	-<br>[4]	-<br>[4]	-<br>[4]	-<br>[4]	-<br>[4]	-<br>[4]	-<br>[4]

	[1]<br>- *KOH/S<br>[1]<br>- *D<br>[1]	[2]<br>- *KOH/S<br>[2]<br>- *D<br>[2]	[2]<br>- *KOH/S<br>[2]<br>- *D<br>[2]	[2]<br>- *KOH/S<br>[2]<br>- *D<br>[2]	[3]<br>- *KOH/S<br>[3]<br>- *D<br>[3]							
**LTA**	-<br>[1]<br>- *S<br>[1]<br>- *KOH/S<br>[1]<br>- *D<br>[1]	-<br>[2]<br>- *S<br>[2]<br>- *KOH/S<br>[2]<br>- *D<br>[2]	-<br>[2]<br>- *S<br>[2]<br>- *KOH/S<br>[2]<br>- *D<br>[2]	-<br>[2]<br>- *S<br>[2]<br>- *KOH/S<br>[2]<br>- *D<br>[2]	-<br>[3]	-<br>[4]	-<br>[4]	-<br>[4]	-<br>[4]	-<br>[4]	-<br>[4]	-<br>[4]
**SNA**						+<br>[4]	+<br>[4]	-<br>[4]	-<br>[4]	+<br>[4]	+<br>[4]	-<br>[4]
**GSA-II**	-<br>[1]<br>- *S<br>[1]<br>- *KOH/S<br>[1]<br>- *D<br>[1]	-<br>[2]<br>- *S<br>[2]<br>- *KOH/S<br>[2]<br>- *D<br>[2]	-<br>[2]<br>- *S<br>[2]<br>- *KOH/S<br>[2]<br>- *D<br>[2]	-<br>[2]<br>- *S<br>[2]<br>- *KOH/S<br>[2]<br>- *D<br>[2]	-<br>[3]<br>- *S<br>[3]<br>- *KOH/S<br>[3]<br>- *D<br>[3]							
**GSA-IB4**	-<br>[1]<br>- *S<br>[1]<br>+ *KOH/S<br>[1]	-<br>[2]<br>+ *S unif.<br>[2]<br>+ *KOH/S<br>[2]<br>+ *D<br>[2]	-<br>[2]<br>+ *S unif.<br>[2]<br>+ *KOH/S<br>[2]<br>+ *D<br>[2]	-<br>[2]<br>+ *S unif.<br>[2]<br>+ *KOH/S<br>[2]<br>+ *D<br>[2]	-<br>[3]<br>+ *S<br>[3]<br>+ *KOH/S<br>[3]							
**ECA**					-<br>[3]<br>+ *S<br>[3]<br>+ *KOH/S<br>[3]	-<br>[4]	+<br>[4]	+<br>[4]	-<br>[4]	-<br>[4]	+<br>[4]	+<br>[4]
**PSA**						-<br>[4]	+<br>[4]	-<br>[4]	-<br>[4]	+<br>[4]	+<br>[4]	-<br>[4]

Analysis were carried out using sections of ovaries.
Positive binding: **+**; Negative binding: -
Binding localized in the external ZP surface: **ext.**; internal ZP surface: **int.**; medium ZP: **med.** or uniform binding: **unif.**
*: lectin binding procedures were preceded by different pre-treatments. Sialidase digestion: **S**; Saponification/sialidase digestion: **KOH/S**; Desulphation process: **D.**
Some references distinguish between primordial, primary, secondary, multilaminar and antral follicles.

References:

1. Parillo, F.; Diverio, S.; Todini, L.; Fagioli, O. Histochemical detection of the lectin-binding carbohydrates in the zona pellucida during oocyte growth in the wild boar "Sus scrofa scrofa." *Vet. Res.* **2001**, *32*, 581–590, doi:10.1051/vetres:2001147.

2. Parillo, F.; Diverio, S.; Romeo, G.; Fagioli, O. Variations in lectin-binding on the zona pellucida during oocyte growth in some wild ungulates. *Ann. Anat.* **2003**, *185*, 109–115, doi:10.1016/S0940-9602(03)80071-7.

3. Parillo, F.; Stradaioli, G.; Verini-Supplizi, A. Glycoconjugates in small antral ovarian follicles of the river buffalo (Bubalus bubalis L.). *Acta Histochem.* **1998**, *100*, 229–243, doi:10.1016/S0065-1281(98)80010-X.

4. Chapman, J.A.; Wiebkin, O.W.; Breed, W.G. Interspecific variation of zona pellucida glycoconjugates in several species of marsupial. *J. Reprod. Fertil.* **2000**, *119*, 111–120, doi:10.1530/jrf.0.1190111.

Table S3. Primers used for the RT-PCR amplification of *GAPDH* and *ZPAX* genes of Bennett's wallaby.

Gene	Forward 5´-3´	Reverse 5´-3´	Product size (bp)	GenBank
GAPDH	ATGCCCCAATGTTCGTGAT	ACAACAGATACATTGGGAGT	349	EF654515.1
ZPAX	CCAAGATGAGCTGAAGACTG	GCATCTTCAACATATCCGTG	720	Scaffold20091 Macropus eugenii
ZPAX	GATACAGTCTGAGGAGTGATG	CACGGATATGTTGAAGATGC	266	Scaffold20091 Macropus eugenii
ZPAX	GCAATGTAGACCAGAGCTG	GGACAAGTGATGGAGTATTC	241	Scaffold20091 Macropus eugenii
